# Primary synovial sarcoma of the kidney: a case report of complete pathological response at a Lebanese tertiary care center

**DOI:** 10.1186/s12894-018-0358-z

**Published:** 2018-05-11

**Authors:** Alissar El Chediak, Deborah Mukherji, Sally Temraz, Samer Nassif, Sara Sinno, Rami Mahfouz, Ali Shamseddine

**Affiliations:** 1 Department of Internal Medicine, Division of Hematology/Oncology, American University of Beirut - Medical Center, P.o.Box: 11-0236, Riad El Solh, Beirut, 110 72020 Lebanon; 2 Department of Pathology and Laboratory Medicine, American University of Beirut - Medical Center, Beirut, Lebanon

**Keywords:** Synovial sarcoma, SYT-SSX, Doxorubicin, Ifosfamide, Pathological response, Survival

## Abstract

**Background:**

Primary synovial sarcoma of the kidney is a rare type of soft tissue sarcoma. Its presenting features can resemble those of other renal tumors; rendering its early diagnosis, a dilemma. Several cases of renal synovial sarcoma have been reported in the literature with varying treatment options and outcomes. This article describes a rare case of primary renal synovial sarcoma and reviews all cases in the literature.

**Case presentation:**

A 26-year-old male presented with flank pain and hematuria. Initially diagnosed with Wilm’s tumor, revision of pathology and histology, along with the immunohistochemical profile, confirmed, nevertheless, the diagnosis of primary monophasic synovial sarcoma of the kidney with the SYT-SSX2 fusion transcript. Follow-up, post nephrectomy, revealed recurrence within the lungs and at the surgical bed. Surgical resection followed by adjuvant chemotherapy regimen constituting of Doxorubicin and Ifosfamide, achieved complete pathological response.

**Conclusion:**

In this case report, we emphasize the need for accurate diagnosis and prompt treatment. We propose multimodality treatment approach including surgery along with anthracycline-based chemotherapy to induce complete remission.

## Background

Soft tissue sarcoma (STS) is a rare malignant tumor of mesenchymal origin having an incidence of 2–3 cases per 100,000, thus contributing to less than 1% of all adult malignancies [1, 2]. Synovial sarcoma (SS), or sarcoma of tissues adjacent to joints, is a rare type of STS, and represent 5 to10% of all STSs [1]. SS is commonly found in the proximal limb of young adults and has a male predominance [3]. Other unusual sites of occurrence include the head and neck, heart, lungs, and kidneys [4]. Very few reports have tackled this tumor due to its rarity and difficulty to distinguish from other renal pathologies. The first case of primary SS of the kidney has been reported by Faria et al. in 1999 [5]. We present a case of primary synovial sarcoma of the kidney, initially thought to be a Wilm’s tumor, along with patient follow-up, showing complete pathological response to treatment, followed by a literature review of this disease entity.

## Case presentation

**Fig. 1:**
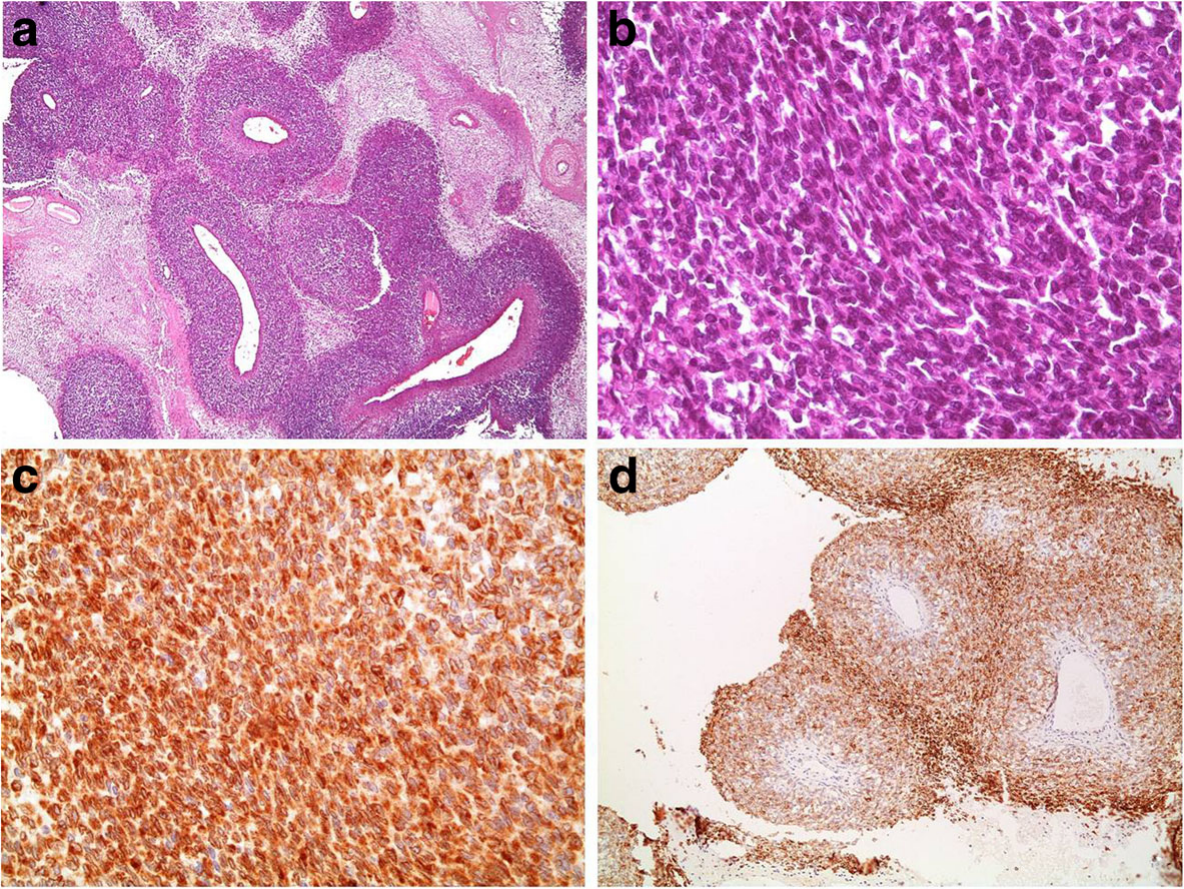
Partially necrotic, densely cellular proliferation with a prominent perivascular growth pattern (**a**, H&E stain, 40×). Tumor cells are essentially spindle in appearance (**b**, H&E stain, 400×), and express vimentin (not shown), focal keratin (not shown), BCL-2 (**c**, 400×), and CD56 (**d**, 100×)

A 26-year-old male experienced recurrent flank pain and gross hematuria over several months duration. Kidney ultrasound showed a lower pole mass concerning for renal cell carcinoma. After confirmation of a right kidney tumor, measuring 6 cm, by an enhanced CT scan, he underwent right radical nephrectomy with para-caval lymph node dissection, at another institute, with pathology there, read initially as adult type Wilm’s tumor. After referral to our institute for rereading of the pathological slides, the morphological and immunostaining profiles were analyzed, and results came out to be consistent with synovial sarcoma of the right kidney. The tumor was monophasic and showed a cellular spindle cell proliferation with a prominent perivascular growth pattern and partial necrosis. It was positive for vimentin, BCL-2, CD56, MCK (partial), and negative for CD10, 31, 34, 99, 117, CK7, Desmin, SMA, MyoD1, EMA, WT-1, S100, RCC, PAX8, GATA-3, and Synaptophysin (Fig. 1).

Molecular studies on the paraffin-embedded blocks were performed to test for the t(X; 18) SYT/SSX fusion transcript, using RT-PCR, at the University of Michigan Health System. RT-PCR amplification was performed using fluorescent dye-labeled primers, specific for the SYT-SS18 and SYT-SSX genes. The PCR products were then detected and sized by capillary electrophoresis to identify the presence of chimeric transcripts. A concurrent internal control was run to ensure the integrity of the mRNA. FISH analysis was also performed using a break-apart style probe. The results were unfortunately negative due to the low quality samples.

According to these findings, a diagnosis of primary monophasic SS of the kidney was made. It was elected for serial follow up and no adjuvant treatment, thereafter. Six months later, a follow up CT scan detected a 1.5cmx1.7 cm left lower lobe lung nodule suggestive of metastasis. Consequently, he underwent a smooth left lower lobe wedge resection. Fusion gene product analysis on the resected lung tissue, via FISH, revealed SYT-SSX 2 gene rearrangement confirming the SS diagnosis. Three months afterwards, CT scan of the chest, abdomen, and pelvis revealed another disease recurrence in the nephrectomy surgical bed, with tumor invasion of the inferior vena cava and the presence of conglomerate suspicious aorto-iliac lymph nodes. A multidisciplinary team approach decided to start the patient on Doxorubicin 50 g/m^2^ and Ifosfamide 5 g/m^2^ chemotherapeutic regimen. Following the third cycle, CT scan and MRI showed a 30 to 50% interval decrease in size of tumor masses in the right nephrectomy bed and adjacent retroperitoneum, IVC tumor, and distal aortocaval lymph nodes, indicating partial treatment response. The patient were received a total of 5 cycles, with no adjunct side effects.

A follow-up MRI, several months later, showed continued decrease in the size of 3 masses at the previous surgical site, IVC tumor invasion, and aortocaval lymph nodes, indicating continued response to treatment. One of the small masses in the nephrectomy bed almost completely resolved, on imaging, with no new progression. It was then decided to have the patient undergo surgical resection of the residual masses at the previous surgical bed with removal of the aorto-caval lymph nodes, thrombectomy with vena caval repair. All surgical margins were negative. Final pathology came out to be necrosis, with no viable tumor identified. Thus, a complete pathological response was achieved using the Adriamycin/Ifosfamide regimen, a year after the initial nephrectomy. A sample of the kidney lysate was again tested for the (X; 18) SYT/SSX fusion transcript via RT-PCR and FISH, and results were negative, suggestive of complete treatment response.

## Discussion

Synovial sarcoma is a mesenchymal spindle cell tumor which displays variable epithelial differentiation and has a specific chromosomal translocation t(X; 18) (p11; q11), which results from the fusion of the SYT gene on chromosome 18 to exon 5 of either SSX1 or SSX2 genes on chromosome X [6]. It was recently reported that the SSX4 gene is also involved in such a translocation [6, 7].Nonetheless, SS of the kidney can be first misdiagnosed as a renal cell carcinoma due to similar clinical presentation [3]. The identification of the monophasic type of renal SS is also controversial as it has similar microscopic features to other spindle cell tumors, such as fibrosarcoma, leiomyosarcoma, malignant peripheral nerve sheath tumors, adult Wilm’s tumor, spindle cell carcinoma and spindle cell melanoma [3]. While monophasic SS of the kidney is made up exclusively of monomorphic spindle cells, the biphasic type is a mixture of both spindle-shaped cells and epithelial cells. SS of the kidney is a rare disease such as 64 cases have been reported since the Faria et al. cases up to 2012 [6]. We conducted a literature review using Embase and PubMed databases and included all cases (even those published in languages other than English) till the year 2016. This yielded a total of 114 cases (Table 1) constituting the largest series of renal SS cases to be reported. Noteworthy, our case is the first to be reported from the Middle East*.*

**Table 1:** List of 114 cases of renal SS published in the literature

Case Report/Series	Author/ Year of publication	No of cases	Age(Y)/Gender (M/F)	Presenting symptoms	Fusion gene Variant	Treatment	Outcome
1	Argani P et al.; 2000 [5]	17	10 M 7 F Median age: 35	Abdominal pain; hematuria; incidental finding for hypertension workup; other data not available	1: SYT-SSX1; 4: SYT-SSX2	Radical nephrectomy	N/A
2	Kim DH et al.; 2000 [17]	2	53/M 47/M	Rt flank pain Rt flank pain, gross hematuria	SYT-SSX2;	Rt radical nephrectomy; Rt radical nephrectomy with IVC thrombectomy	No recurrence 6 mons later; lung mets 5 mons later, death 10mons post-op
3	Chen S et al.; 2001 [18]	1	48/M	Hematuria	SYT-SSX 2	Lt radical nephroureterectomy; Radiation to surgical bed; 4 cycles of ifosfamide and Doxorubicin	N/A
4	Koyama S et al.; 2001 [8]	1	47/F	Right back pain	SYT-SSX 2	Rt radical nephrectomy	No recurrence 17 mons later
5	Bella AJ et al.; 2002 [25]	1	24/M	Gross hematuria	SYT-SSX t(X;18)	Rt radical nephrectomy, adjuvant Actinomycin + Vincristine	No clinical evidence of disease 18 mons after nephrectomy
6	Dai YC et al.; 2002 [26]	1	19/F	Abdominal Pain, 3 mons of amenorrhea	SYT-SSX t(X;18)	Rt nephrectomy	Recurrence of tumor in retroperitoneum and abdominal wall 9 mons after surgery
7	Vesoulis Z et al.; 2003 [27]	1	38/M	Acute abdominal pain	SST-SSX1	Lt radical nephrectomy	N/A
8	Moch H et al.; 2003 [28]	2	47/M 56/F	Renal mass	SST-SSX1/ SYT-SSX2	Nephrectomy/ Rt nephrectomy	Local recurrence 11 years later/ N/A
9	Chen PC et al.; 2003 [29]	1	19/M	left flank pain and intermittent hematuria	N/A	Lt radical nephrectomy + IVC thrombectomy; adjuvant Ifosfamide + Etoposide	died of sepsis 1 month after surgery
10	Park SJ et al.; 2004 [9]	1	32/F	Intermittent abdominal pain	N/A	Lt radical nephrectomy + thormbectomy; 6 cycles Ifosfamide + Doxorubicin	Metastasis to lung 4 mons post-op, complete remission after chemotherapy
11	Jun SY t al; 2004 [30]	3	27, 35/F 26/M	Rt flank pain	SYT-SSX2	Rt radical nephrectomies	1F: disease free 5 mons post-op; 2F: lumbar vertebral mets 5 mons post-op, 6 mons disease free post-resection; 3 M: bilateral hemothorax; death 34 days post-op
12	Tornkvist M et al.; 2004 [24]	1	34/F	N/A	SYT-SSX 2	Rt nephrectomy, chemotherapy	Visceral recurrence; lung metastases
13	Schaal CH et al.; 2004 [14]	1	27/M	Hematuria and large abdominal mass	N/A	Ifosfamide and Adriamycin, followed by Rt Radical nephrectomy	No recurrence after one year
14	Shao L et al.; 2004 [31]	4	N/A	N/A	N/A	N/A	N/A
15	Shannon BA et al.; 2005 [32]	1	60/M	Hematuria	SYT-SSX 2	Rt radical nephrectomy, Imatinib, 5 cycles of adjuvant chemotherapy	Pulmonary metastasis 6 mons after surgery; death 12 mons later
16	Perlmutter AE et al.; 2005 [33]	1	61/F	Right flank pain and gross hematuria	SYT-SSX 2	Rt nephrectomy, refused adjuvant chemotherapy	No recurrence 5 mons post-surgery
17	Stage et al.; 2005 [34]	1	51/F	Renal masses incidentally found	N/A	N/A	N/A
18	Paláu L MA et al.; 2007 [35]	1	71/F	Flank pain and gross hematuria	SYT-SSX 2	Lt nephrectomy	Recovery in 22 mons after surgery
19	Drozenova et al.; 2008 [36]	2	33/M 57/F	Rt flank pain Lt flank pain	SYT-SSX1/ SYT-SSX1	Rt Radical nephrectomy/ Lt Radical nephrectomy	Local recurrence and lung mets; death 6 mons later/ N/A
20	Mirza M et al.; 2008 [37]	1	17/M	Flank pain and gross hematuria	SYT-SSX 2	Lt radical nephrectomy	No recurrence 1 year later
21	Gabilondo F et al.; 2008 [38]	1	32/F	Mild abdominal pain; gross hematuria	SYT-SSX t(X;18)	Rt radical nephrectomy	N/A
22	Zakhary MM et al.; 2008 [39]	1	52/F	Right flank pain	N/A	Rt nephrectomy	N/A
23	Chung SD et al.; 2008 [40]	2	30/F 49/F	Rt flank pain Lt loin pain	SYT-SSX1	Rt radical nephrectomy/ Lt radical nephrectomy	No recurrence 15 mons post-op/ No recurrence 27 mons post-op
24	Erturhan S et al.; 2008 [41]	1	59/M	Right lumbar pain and palpable mass	N/A	N/A	N/A
25	Divetia M et al.; 2008 [42]	7	2-M 5-F (15–46 years)	Abdominal lump, hematuria	3 SYT-SSX1/ 1 SYT-SSX2	Radical nephrectomy	Lung mets in 2 patients; death at 6 and 12 mons, respectively
26	Dassi V et al.; 2009 [43]	1	20/F	Flank pain	SYT-SSX t(X;18)	Lt radical nephrectomy	N/A
28	Kawahara et al.; 2009 [44]	1	40/F	Abdominal pain	SYT-SSX 1	Radical nephrectomy	N/A
29	Long JA et al.; 2009 [45]	3	(Age range: 27–33 years)	Back pain and spontaneous rupture	SYT-SSX t(X;18)	2 Rt radical nephrectomy; 1 Lt radical nephrectomy	2 patients: total remission 25 mons post-op; 1 patient: death 24 mons post-op
30	Wezel F et al.; 2010 [46]	1	47/M	Hematuria, abdominal pain, weight loss	SYT-SSX t(X;18)	Nephrectomy	No recurrence 18 weeks after surgery
31	Wang Z-H et al.; 2009 [47]	4	2/F 2/M 32 to 48 years	Low back pain, hematuria	SYT-SSX1	Radical nephrectomy; 3 Lt side, 1 Rt side	Liver + lung metastasis; death at 5, 8, 18, and 21 mons post-op, respectively
32	Kageyama S et al.; 2010 [48]	1	67/M	Gross hematuria and right flank pain	SYT-SSX 2	Rt nephroureterectomy; Ifosfamide and Etoposide regimen	Tumor recurrence 33 mons post nephrectomy; Liver mets; death 4 years later
33	Tan YS et al.; 2010 [49]	4	N/A	N/A	N/A	N/A	N/A
34	Romero-Rojas AE et al.; 2013 [50]	1	15/M	Lt abdominal pain; weight loss	N/A	Neoadjuvant chemotherapy followed by Lt radical nephrectomy	Death 1.8 years later
35	Lakshmaiah KC et al.; 2010 [51]	2	50/F 45/M	Rt flank pain/ Lt flank pain, hematuria	SYT-SSX2/ NOT DONE	Radical nephrectomy	No recurrence 2 years post-op/ lost to follow-up
36	Kataria et al.; 2010 [52]	1	52/F	Renal mass	SYT-SSX 2	Radical nephrectomy IVC thrombectomy; adjuvant chemo-radiation	Mets to lung
37	Grampurohit VU et al.; 2011 [53]	1	21/F	Fever, hematuria; right flank pain	SYT-SSX t(X;18)	Rt nephrectomy	No recurrence 6 mons post-surgery
38	Ozkan EE et al.; 2011 [20]	1	68/F	Right flank pain and abdominal distention	N/A	Rt nephroureterectomy, 4 cycles Ifosfamide and Doxorubicin	No recurrence one year later
39	Karafin M et al.; 2011 [54]	3	39/F 41/M 53/M	N/A	SYT-SSX2 SYT-SSX2 N/A	N/A	N/A
40	Nishida T et al.; 2011 [55]	1	63/F	Dysuria, hematuria	SYT-SSX 1 & 2	Rt Radical nephrectomy	No recurrence one year postop
41	Pitino A et al.; 2011 [8]	1	67/M	Lumbar pain, gross hematuria	SYT-SSX 2	Lt Nephroureterectomy; adjuvant Epirubicin post-op	Local recurrence of disease 24 mons post- surgery
42	Bakhshi et al.; 2012 [56]	1	33/F	Abdominal pain and gross hematuria	SYT-SSX 2	Lt radical nephrectomy; external radiotherapy	No recurrence at 2 years
43	Lopes et al.; 2013 [3]	1	19/M	Lumbar pain, gross hematuria	Negative translocation	Lt nephrectomy, thrombectomy; 5 cycles of doxorubicin	Lung mets several mons post-op
44	Pereira E Silva R et al.; 2013 [57]	1	17/M	Incidental large renal mass after workup for secondary hypertension	Negative translocation	Radical nephrectomy followed by ifosfamide	No recurrence 29 mons later
45	Marković-Lipkovski J et al.; 2013 [12]	1	38/M	Rt flank pain; fever; hematuria	SYT-SSX2	Rt radical nephrectomy	Died three mons later
46	Moorthy et al.; 2014 [58]	1	46/M	Flank pain	SYT-SSX 2	Lt radical nephrectomy	N/A
47	Majumber et al.; 2014 [59]	1	46/F	Flank pain, hematuria	N/A	Rt radical nephrectomy	No evidence of disease after 2 mons follow up
48	Schoolmeester JK et al.; 2014 [60]	16	9M/7F 17-78 yrs.; Median: 46 yrs	N/A; Rt: 10; Lt: 6;	SYT-SSX2: 10; SYT-SSX1: 5; 1: failed	14: Radical nephrectomy; 1: partial nephrectomy; 1: needle biopsy	6: death within 1–58 mons (mean 31mons); 5: no recurrence 12–77 mons (39 mons); 1: alive with spine mets 11mons later
49	Kim MS et al.; 2014 [61]	1	38/F	Lt flank pain	SYT-SSX2	Lap Lt radical nephrectomy followed by radiation to surgical bed	Recurrence at the distal ureter and uretero-vesical junction 6 mons post- surgery
50	Ozkanli SS et al.; 2014 [62]	1	45/M	Flank pain; macroscopic hematuria	SYT-SSX t(X;18)	Lt radical nephrectomy	N/A
51	Mishra S et al.; 2015 [13]	1	60/M	Flank pain, hematuria	SYT-SSX t(X;18)	Radical nephrectomy	N/A
52	Wang Z et al.; 2015 [61]	1	54/F	Flank pain, hematuria	SYT-SSX 1	Radical nephrectomy	No recurrence 12 mons post-surgery
53	Vedana M et al.; 2015 [63]	1	76/F	Flank pain; hematuria	SYT-SSX t(X;18)	Rt radical nephro-ureterectomy	No recurrence 20 mons post-surgery
54	Lv X-F et al.; 2015 [64]	5	2F/3M (15–43 yrs.; Median: 27.4 yrs)	N/A	N/A	N/A	N/A
55	Present case El Chediak A. et al.; 2016	1	26/M	Rt flank pain; hematuria	SYT-SSX2	Rt radical nephrectomy, Doxorubicin Ifosfamide	Lung metastasis 6 mons post nephrectomy; no recurrence one year post chemotherapy

*M* Males, *F* Females, *Yrs* Years, *Rt* Right, *Lt* Left, *Mets* Metastasis, *Mons* Months, *IVC* Inferior vena cava, *N/A* Not Applicable

The median age of patients with renal SS was 40.5 (15–78) years, which is half the median age for diagnosis of renal cell carcinoma [7]. The female to male ratio was 1:1. Regarding the predominant presentation symptoms, data was only available for 82 cases. The most frequently reported symptom on presentation was isolated flank/lumbar pain which was found in 20 patients (24.4%).This is in concordance with a review conducted on older data where this same predominant symptom occurred in 55.5% of cases [8]. Hematuria was present in 37 patients (44%) upon presentation. Kohle et al. reported similar data, albeit smaller sample size, where 98% of their patients were symptomatic at the time of presentation, with 67% having pain and 38% having hematuria [6]. These figures are in concordance with our analysis of the world literature.

According to our analysis, the leading fusion variant was the SYT-SSX 2, detected in 42 (36.8%) patients, as opposed to the SYT-SSX 1 variant, detected in 23 (20.2%) patients, in total (Table 1).

Data on metastasis and disease recurrence was available for 70 and 57 patients, respectively. 19 (27.1%) patients had metastasis, whereas 14 (24.6%) patients had tumor recurrence on follow-up. These numbers are similar to what was published in previous series [9].

A 30 to 50% of the patients who underwent surgical resection of their primary tumor were reported to witness metastasis to the lungs or liver [10]. Our patient is of no exception. In terms of median survival for patients with localized disease, size represents an important prognostic factor [10]. A retrospective analysis was done on 135 consecutive patients with extremity and truncal variant of synovial sarcomas, seen at three institutions in Boston, between years 1961 and 1996. Patients with localized synovial sarcomas, less than 5 cm in longest diameter, had a survival at 10 years equivalent to 88%, compared with a 10-year survival of 38 and 8% for 5 to 10 cm and greater than 10 cm sarcomas, respectively [10]. Similar results were reported by Singer et al. on 48 consecutive patients with extremity and truncal synovial sarcomas, seen between years 1966 and 1994 [11].

SS of the kidneys presents a diagnostic dilemma, as it resembles renal cell carcinoma amongst other tumors. Diagnosis can be based on morphological (spindle cell) and immunological tumor profile. It can also be established by genetic analysis via FISH and RT-PCR, demonstrating the SYT18-SSX gene translocation. Demonstrating which translocation, the tumor possesses, has been a delicate matter. For instance, a SS case that was previously shown to be negative for SYT/SSX1 and SYT/SSX2 gene expression by conventional RT-PCR, was instead found to be SYT/SSX4 positive, as the sole fusion transcript expressed in this tumor sample, when the RT-PCR was redesigned [6]. This might have explained our negative results, when trying to identify the translocations, since our RT-PCR was only designed to detect SYT/SSX1 and SYT/SSX2 fusion gene variants.

Moreover, the value of TLE1 antibody in the diagnosis of SS has also been examined. TLE1 was found to be an excellent discriminator of SS from other sarcomas, in a study by Terry et al. [12]. They reported that TLE1 monoclonal and polyclonal antibodies gave intense and/or diffuse nuclear staining in 91 out of 94 molecularly confirmed synovial sarcoma patients. In contrast, TLE1 staining has been detected much less frequently and at lower levels, if et al.l, in 40 other mesenchymal tumors; thereby making this a robust immunohistochemical marker for SS. Jagdis et al. also supported this view as their findings confirmed that TLE1 was more sensitive and specific for synovial sarcoma, than any other currently available immunohistochemical kits [13]. Kosemehmetoglu et al. confirmed the sensitivity, but not the specificity, of TLE1 antibodies in diagnosing synovial sarcomas [14].

The prognostic implication of SYT-SSX fusion type in synovial sarcomas is still under debate. The SYT-SSX fusion type and the presence of metastasis, at diagnosis, were both proven to be important prognostic indicators [15]. Kawai et al. analyzed SYT–SSX fusion transcripts in 45 synovial sarcomas by reverse-transcriptase polymerase chain reaction, and compared the results with relevant clinical and pathological data [16]. SYT- SSX2 fusion type carried a significant positive prognosis for overall survival [16]. This was thought to be due to an association with a lower prevalence of metastatic disease at diagnosis, in patients having this rearrangement [16]. In another study by Ladanyi et al., the SYT–SSX2 fusion transcript had a significantly longer metastasis-free survival [15]. On the contrary, Japanese patients, with synovial sarcoma, having positive SYT-SSX fusion transcript, were retrospectively analyzed [17]. They concluded that *SYT-SSX* fusion type was not found to be a significant prognostic factor, unlike tumor size and histological grading, for patients with localized synovial sarcoma [17]. Another study by Guillou et al. also confirmed that histologic grading, and not SYT-SSX fusion type, was a stronger predictor of survival, by collecting retrospective data on 165 SS patients [18].

In our sample, staging information was available for 46 patients, based on the 7th edition TNM staging for soft tissue sarcomas. Among the patients having the SYT-SSX2 fusion protein. 53.8% were stage II and 34.6% were of stage III. 33 and 25% of patients with SYT-SSX1 transcript were stage II and III, respectively. 25% of patients with SYT-SSX1 were of stage IV, versus only 7.7% for SYT-SSX2 patients.

Lungs were the most common metastatic site, regardless of the fusion type. However, 50% of patients with SYT-SSX2 fusion type had metastasis to the liver. Although lungs and liver are common sites for metastasis for renal SS [19, 20], it was not reported before whether there is a relation between the site of metastasis and the type of fusion transcript. From the above, it appears that SYT-SSX 1 behaves more aggressively. However, studies with a larger number of patients and longer follow-up periods are needed to verify these observations, especially in the light of the contradicting data, presenting on the prognostic value of the SYT-SSX fusion protein.

Although SS is considered an aggressive form of STS where metastasis can occur in 50% of the cases, it was found to be sensitive to Anthracycline based chemo-therapy [21]. However, due to the rarity of the tumor, a standard therapy has not been established. Treatment modalities include surgical resection and chemotherapy. A combination of chemotherapy (Ifosfamide and Doxorubicin) and surgery has yielded positive results [6, 9, 12, 13]. Based on our review of the literature, 10 patients took Ifosfamide and Doxorubicin, either together or in combination with other chemotherapeutic agents. 5 out of 10 cases were reported to have complete remission. This further corroborates the effectiveness of giving Ifosfamide and Doxorubicin as a regimen to treat primary renal SS. The basis for chemotherapy was tumor volume reduction, mainly attributed to Ifosfamide. In one case report, the combination of Ifosfamide and Doxorubicin lead to a 50% reduction of the tumor before consequent resection [14]. The controversy of the impact of adjuvant chemotherapy on overall survival, in SS patients, is limited by randomized clinical trials’(RCTs) sample size and varied chemotherapy regimens with discrepant results [22]. The Sarcoma Meta-analysis Collaboration (SMAC) group performed a meta-analysis of all known randomized clinical trials in 1997. Their results indicated that doxorubicin-based chemotherapy served to significantly improve time to local and distant recurrence, as well as overall recurrence-free survival in comparison to patients who were just observed [23]. An increase in overall survival was not statistically significant [23]. Another meta-Analysis of RCTs of adjuvant chemotherapy for localized resectable STS was conducted by Pervais et al. where they built on the results of the SMAC study and narrowed the confidence intervals [24]. This meta-analysis demonstrated marginal efficacy of doxorubicin based chemotherapy with respect to local recurrence, distant recurrence, overall recurrence, and overall survival, in comparison to those who did not receive adjuvant chemotherapy [22].

## Conclusion

In conclusion, primary SS of the kidney is an aggressive rare disease that can be mistaken for other types of renal cell carcinomas. Its diagnosis is based on morphological and molecular studies demonstrating spindle cells and the SYT-SSX translocation. However, establishing a correct diagnosis may be difficult. Prognosis can be enhanced by use of anthracycline based chemotherapy. Moreover, the combination of surgery and chemotherapy has shown positive results. Particularly, we propose the use of Ifosfamide and Doxorubicin as a standard chemotherapy to induce complete remission. Since the disease may have rapid course with unfavorable outcomes, clinicians need to be aware of the existence of this rare entity, so that timely and appropriate therapy can be initiated.

## Abbreviations

CT: Computer tomography
FISH: fluorescence in situ hybridization
IVC: Inferior vena cava
MRI: Magnetic resonance imaging
N/A: Not available
RCT: Randomized clinical trials
RT-PCR: reverse transcriptase-polymerase chain reaction
SS: Synovial sarcoma
STS: Soft tissue sarcoma
